# Life expectancy in ants explains variation in helpfulness regardless of phylogenetic relatedness

**DOI:** 10.1093/beheco/arae104

**Published:** 2024-12-17

**Authors:** Filip Turza, Daniel Stec, Diego Fontaneto, Krzysztof Miler

**Affiliations:** Doctoral School of Exact and Natural Sciences, Jagiellonian University, prof. S. Łojasiewicza 11, 30-348 Kraków, Poland; Institute of Environmental Sciences, Faculty of Biology, Jagiellonian University, Gronostajowa 7, 30-387 Kraków, Poland; Institute of Systematics and Evolution of Animals, Polish Academy of Sciences, Sławkowska 17, 31-016 Kraków, Poland; Molecular Ecology Group (MEG), National Research Council of Italy, Water Research Institute (CNR-IRSA), Largo Tonolli 50, Verbania Pallanza, Italy; National Biodiversity Future Center (NBFC), Piazza Marina 61, Palermo, Italy; Institute of Systematics and Evolution of Animals, Polish Academy of Sciences, Sławkowska 17, 31-016 Kraków, Poland

**Keywords:** altruism, Formicidae, lifespan, pro-social behavior, rescue behavior

## Abstract

Rescue behavior aims to free a relative from danger. Ants are particularly known for such helpfulness and, perhaps not coincidentally, also show the highest level of social organization in the animal kingdom, i.e. eusociality. However, even among social species such as ants, there is a huge variation in rescue proneness, and little is understood about the underlying causes of this variation. In this study, we explore the relationship between helpfulness in the form of rescue and life expectancy, focusing on 14 ant species with diverse phylogenetic backgrounds. We posit that species with longer worker life expectancies are more prone to engaging in rescue actions. To test this, we assessed worker lifespan in each species and conducted behavioral tests simulating entrapment scenarios involving a nestmate ensnared by an artificial obstacle. Observed behaviors involved contact with the nestmate, digging around it, pulling at its body parts, and biting the entrapping obstacle. Our findings reveal that species with longer worker life expectancies exhibit higher proneness to rescue endangered nestmates, irrespective of phylogenetic relatedness. Furthermore, we found no trace of a phylogenetic signal in the life expectancies or helpfulness of workers belonging to different species. The results underscore the significance of life expectancy as a key factor influencing the likelihood of rescue behavior in ants. This phenomenon warrants further investigation, given the varied physiologies, life histories, and ecologies observed among species. Nevertheless, the impact of life expectancy on behavioral patterns in social insects suggests that this parameter is likely significant across diverse taxa.

## Teaser

Rescue behavior in ants involves helping endangered nestmates. Our study examined several ant species, finding that those with longer worker lifespans were more likely to engage in rescue actions. This suggests life expectancy is a crucial factor influencing rescue behavior. These findings highlight the need for further research on the behavioral patterns of social insects.

## Introduction

Pro-sociality involves individuals voluntarily benefiting a group through their actions (Hamilton 1963). These actions impose unavoidable costs on pro-social individuals (Decety and Svetlova 2012; Decety et al. 2016). Given the role of pro-sociality in promoting group survival (de Waal 2008), it is prevalent in social organisms (Pennisi 2005). Our need for understanding pro-social behavior in non-human species is strongly marked in the scientific literature (e.g. Cronin 2012; Marshall-Pescini et al. 2016; Chen et al. 2023; Nafcha et al. 2023). A type of pro-sociality that has garnered special attention is rescue. During rescue, one individual (the rescuer) provides help to another individual (the victim) in a dangerous situation (Nowbahari and Hollis 2010). This altruistic act carries the potential risk of injury or even death for the rescuer (Vasconcelos et al. 2012) at no immediate advantage to that particular individual (Nowbahari et al. 2016). Rescue actions seem widespread across species. They have been observed in various taxa, including birds (Hammers and Brouwer 2017; Crampton et al. 2022), elephants (Bates et al. 2008), primates (e.g. Vogel and Fuentes-Jiménez 2006; Huang et al. 2020), rodents (Bartal et al. 2011; Ueno et al. 2019), whales (Siebenaler and Caldwell 1956; Pitman et al. 2017), wild boars (Masilkova et al. 2021), and pigs (Moscovice et al. 2023). The rescue has been documented not only in vertebrates but also in ants (reviewed in Miler and Turza 2021; Hollis and Nowbahari 2022), making it a general topic in research devoted to social interactions across various organisms, including both vertebrates and invertebrates (Nowbahari et al. 2016).

Aging is the most widespread and inevitable phenomenon in biology (Cohen et al. 2022). How the aging process impacts the life-history traits and behavioral characteristics of living organisms is a problem long-present in the scientific literature (e.g. Medvedev 1990; Viña et al. 2007; Siracusa et al. 2022). Investigating this problem is difficult because in many cases, age is a worse measure of longevity than life expectancy. Although age strongly correlates with life expectancy (i.e. increasing age decreases life expectancy), the latter may be shaped by factors other than age. For example, it can decrease in organisms exposed to higher risks of diseases, parasite infestation, and predation, or in those more fecund (e.g. Zwaan 1999; Stearns et al. 2000; Chapuisat and Keller 2002). The issue is particularly complex in social insects (Heinze and Giehr 2021; Korb and Heinze 2021). The evolution of eusociality is associated with a 100-fold increase in life expectancy (Keller and Genoud 1997), suggesting a co-evolution between insect sociality and extended longevity (Carey 2001). Indeed, it is thought that more advanced social behaviors provide insurance-based survival advantages, becoming one of the main drivers of extended life expectancy. Nevertheless, although social species may live longer lives than their nonsocial relatives, life expectancies of the former still strongly differ for many reasons, e.g. differences in ecological niches, parasite, disease, and/or predation pressures (Hölldobler and Wilson 1990).

Ants (Hymenoptera: Formicidae) are characterized by eusociality, the highest level of social organization in the animal kingdom (Hölldobler and Wilson 1990). Rescue likely contributes to ants’ ecological and evolutionary success (Andras et al. 2020). The earliest recorded instance of army ants (*Eciton hamatum* (Fabricius, 1782)) attempting to help nestmates trapped beneath stones dates back to the 19th century (Belt 1874). Subsequent studies have provided evidence of ant rescue actions in other situations, such as entrapment under the soil (Markl 1965; Spangler 1968; Hangartner 1969), capture by predatory antlions and spiders (Czechowski et al. 2002; Uy et al. 2019), or after injuries inflicted by termites (Frank et al. 2017). Rescue offers significant fitness advantages to ants by enabling them to respond to threats and save fellow group members. For example, Frank et al. (2017) found that without assistance, 32% of injured termite-eating ants (*Megaponera analis* (Latreille, 1802)) would perish, but rescue mitigated these losses, resulting in a 28.7% increase in colony size. Kwapich and Hölldobler (2019) observed that harvester ants (*Veromessor pergandei* (Mayr, 1886)), after aiding nestmates entangled in spider silk, removed the remaining webs, reducing the risk of other colony members getting ensnared. This is vital for these ants, as losing just 5 foragers per day would equate to a loss of more than 65 500 seeds annually (Kwapich and Hölldobler 2019). These studies highlight the broader benefits of rescue actions for the entire ant colony. However, rescue is considered costly and risky (Nowbahari and Hollis 2010; Duhoo et al. 2017). For example, Kwapich and Hölldobler (2019) estimated that 6.3% of *V. pergandei* foragers engaged in web removal are killed by spiders. These costs may influence the variability in rescue occurrence among ant species (e.g. Czechowski et al. 2002; Hollis and Nowbahari 2013; Miler et al. 2017a; Andras et al. 2020). However, our understanding of the proximate and ultimate causes of differences in helpfulness between species remains limited (Hollis and Nowbahari 2022). Here, we integrate the issues of pro-social helpfulness toward group members and life expectancies in different species of ants. We test how ants with varying worker lifespans differ in their behavioral traits, particularly rescue behavior. A recent study has shown that ant species with different mortality rates might vary in such traits as aggression levels or activity timing (Kwon et al. 2022). Yet, the potential for an across-species pattern in the pro-sociality of ants depending on their life expectancies remains unexplored. Different species of ants are characterized by different rescue proneness (Hollis and Nowbahari 2013; Miler et al. 2017a) and ant species exhibit significant variation in life expectancy even among closely related species (Hölldobler and Wilson 1990; Keller and Genoud 1997; Kramer and Schaible 2013). While there have been suggestions that life expectancy may influence rescue actions within species (e.g. Miler 2016; Miler et al. 2017b; Frank et al. 2018; Turza and Miler 2023), this has not been explored on a larger, cross-species scale. In this study, we explore whether worker life expectancy among 14 ant species representing diverse phylogenetic backgrounds is related to their pro-social behavior in the form of rescue. Our primary hypothesis is that rescue is more likely in species that are characterized by longer-lived workers because helpfulness in groups with high mortality of individuals would probably be less productive.

## Materials and methods

### Field site and ant species

We gathered ants belonging to 14 species and from colonies situated in Lesser Poland, spanning a latitude range of 49°03ʹ to 51°29ʹ and a longitude range of 18°12ʹ to 23°24ʹ (Table 1). The criteria for selection were the commonness of the species, ease of its acquisition, relatively large colonies, and no permits required for collection (Czechowski et al. 2012). We picked 5 distinct colonies of each species to gather relatively reliable species-level data. To ensure uniformity in the individuals tested, we only collected ants found outside the nest, the so-called foragers, also indicated as most rescue-prone among ant castes (Nowbahari et al. 2012). Furthermore, we timed our collections to coincide with the peak of colony development and the highest activity among individuals, typically occurring during the period of nuptial flights, which spans from April to September, depending on the species (Table 1). Approximately 200 foragers from each colony were collected before the mating flights, i.e. when the sexual individuals were still present in the ant colony, and placed in plastic containers along with some nest material. Subsequently, the ants from each colony were transported to the laboratory. Species identification was carried out following Czechowski et al. (2012).

**Table 1.** Ant species within the Formicidae family used in the study, with specified subfamilies, tribes, and dates of nuptial flights, which indicates the points of highest colony development and the time of worker collection in the current study (based on Czechowski et al. 2012).

Subfamily	Tribe	Species	Nuptial flights
Dolichoderinae	Dolichoderini	*Dolichoderus quadripunctatus* (Linnaeus, 1771)	July
Myrmicinae	Myrmicini	*Myrmica rubra* (Linnaeus, 1758) *Myrmica rugulosa* (Nylander, 1849) *Manica rubida* (Latreille, 1802)	August–September August–October April & August–September
	Crematogastrini	*Tetramorium caespitum* (Linnaeus, 1758)	June–August
Formicinae	Formicini	*Formica fusca* (Linnaeus, 1758) *Formica cinerea* (Mayr, 1853) *Formica cunicularia* (Latreille, 1798) *Formica sanguinea* (Latreille, 1798)	July–August July–August August July–August
	Lassini	*Lasius niger* (Linnaeus, 1758) *Lasius emarginatus* (Olivier, 1792) *Lasius brunneus* (Latreille, 1798) *Lasius fuliginosus* (Latreille, 1798) *Lasius umbratus* (Nylander, 1846)	July–August July–August June–July May–October July–September

### Rearing conditions

Ants obtained from each colony were separately housed in a plastic container measuring 40 × 30 × 10 cm. The inner walls of each container were covered with fluon (Sigma-Aldrich, Germany) to prevent escape attempts. The laboratory conditions were maintained at a constant temperature of 24 °C, relative humidity ranging from 40% to 60%, and a 12-h day and night cycle. The ants were provided with an ad libitum supply of water and a Bhatkar diet rich in protein, carbohydrates, vitamins, and minerals, as recommended for many ant species by Czechowski and Pisarski (1992). The ants were acclimated to the laboratory conditions for 2 d before any subsequent procedures. This acclimatization period aligns with established protocols (Miler and Kuszewska 2017; Turza and Miler 2022).

### Survival experiment

After acclimation, we took 50 individuals from each colony to create a separate survival group for the life expectancy analysis. These ants were housed in plastic containers measuring 18 × 15 × 7 cm. The interior walls were treated with fluon (Sigma-Aldrich, Germany) to prevent the escape of animals. The laboratory conditions provided mirrored those described above (see the section Rearing conditions). The ants had access to ad libitum water and the abovementioned diet throughout the experiment. We monitored mortality daily until day 250 since the establishment of each survival group. We analyzed the mortality of a total of 3,500 individuals (14 species × 5 colonies × 50 individuals = 3,500 individuals).

### Behavioral experiment

The individuals remaining after the establishment of groups for the survival experiment were designated for the behavioral experiment. We performed standard laboratory simulations of entrapment (Nowbahari et al. 2009), the most widely used method for studying rescue behavior in ants. In each trial, an individual ant, designated as the victim, was tied using nylon thread passed over the petiole to a piece of filter paper and placed inside the circular arena (diameter: 7 cm, height: 8 cm), partially filled with dry sand. This arrangement ensured that the victim’s body remained visible while the filter paper was concealed beneath a thin layer of sand. Following this, a second ant, designated as the potential rescuer, was introduced into the testing arena. The test was immediately initiated, with each lasting 5 min. All tests were camera recorded using Sony HDR-CX625 cameras positioned directly above the testing arenas. The tests were between 8 AM and 6 PM on a single day for each colony, always in the abovementioned laboratory conditions. Until tests, the ants had constant access to ad libitum water and food in their containers. We performed 50 tests for each colony, amounting to 3,500 dyadic tests conducted (14 species × 5 colonies × 50 tests = 3,500 tests).

### Analysis of the recordings

The tests amounted to a total of 292 h of recordings. To quantify behaviors exhibited, we measured their duration (in seconds). The analysis was performed using the BORIS software v. 7.9.19 (Friard and Gamba 2016). We adhered to a standardized ethogram across species (Table 2). For rescue to occur, contact between the victim and the rescuer is necessary and often quite prolonged. Thus, the measured behaviors included contact, digging around the victim, pulling the victim’s body parts, transporting the sand covering the victim, and biting the nylon thread. Due to the very low frequency of occurrence, we excluded sand transport as a category before proceeding to formal data analysis.

**Table 2.** An ethogram of behaviors that were assessed in the study (based on Hollis and Nowbahari 2013). Sand transport was excluded from analysis in the current study due to very low occurrence.

Behavior	Operational definition
Contact	The ant makes physical contact with the victim’s body, using its antennae
Digging	The ant positions itself close to the victim and pushes sand backwards using its legs
Pulling	The ant grabs the victim’s body and moves it in a backward direction, using its mandibles
Sand transport	The ant grabs a nearby pebble and transports it away from the victim, using its mandibles
Thread biting	The ant bites the nylon thread that ensnares the victim, using its mandibles

### Phylogenetic analysis

To allow a reliable comparison of the effect of life expectancy on rescue between species, we used phylogenetic comparative methods to account for the confounding factor of potential phylogenetic autocorrelation between the 2 analyzed traits. Phylogenetic relationships were thus obtained as a backbone for the phylogenetic comparative models using DNA from the same animals used for the behavioral analyzes. Two individuals from each species were taken for DNA sequencing. Genomic DNA was extracted from whole individuals using Syngen DNA Mini Kit (Syngen Biotech, Poland) following the manufacturer protocol. Using PCR reaction, we amplified 2 DNA fragments, one nuclear (28S rRNA gene) and one mitochondrial (COI). For COI amplification, we used the following primers: LCO1490-JJ (5ʹ-CHACWAAYCATAAAGATATYGG-3ʹ), HCO2198-JJ (5ʹ- AWACTTCVGGRTGVCCAAARAATCA-3ʹ) (Astrin and Stüben 2008). To amplify 28S rRNA, we designed primers de novo based on several ant 28S rRNA gene sequences from GenBank. The primers were as follows: 28S_ant_F1 (5ʹ-CAAGACGGGTCCTAAGAGTACC-3ʹ), 28S_ant_R1 (5ʹ- CAAGACGGGTCCTAAGAGTACC-3ʹ). PCR cocktails, profiles, sequencing reactions, and all respective product cleanings followed the pipeline provided by Stec et al. (2020). Sequencing products were read with the ABI 3130xl sequencer (Genomed, Poland). Sequences were processed in BioEdit v. 7.2.5 (Hall 1999), checked in BLAST (Altschul et al. 1990) to confirm species IDs, and submitted to GenBank (Supplementary Material 1). *Paraponera clavata* (Fabricius, 1775) served as an outgroup.

All obtained sequences represented a single haplotype for each species thus, for phylogenetic analysis, we used only sequences of one specimen per species. The sequences were aligned using the default settings (in the case of COI) and the Q-INS-I method (in the case of the 28S rRNA gene) of MAFFT v. 7 (Katoh et al. 2002; Katoh and Toh 2008) and manually checked against nonconservative alignments in BioEdit. Then, the aligned sequences were trimmed to 685 bp for the 28S rRNA gene and 658 bp for the COI gene. All COI sequences were translated into protein sequences in MEGA11 (Tamura et al. 2021) to check against pseudogenes. The sequences were then concatenated using SequenceMatrix (Vaidya et al. 2011) and appropriate models of sequence evolution, as well as the best partitioning scheme, were chosen using PartitionFinder v. 2.1.1 (Lanfear et al. 2016) under the Akaike Information Criterion (AIC). The ultrametric trees were calculated in BEAST v. 2.6 (Bouckaert et al. 2019). Parameters in BEAST were set as default, except for appropriate models from PartionFinder2, relaxed clock log normal, 100,000,000 generations with tree samples in each 10 000 generation. Three independent BEAST analyses were run through the CIPRES online portal (Miller et al. 2010). The program Tracer v. 1.6 (Rambaut et al. 2014) was then used to ensure Markov chains had reached stationarity and to determine the correct “burn-in” for the analysis which was the first 10% of generations. The ESS values were greater than 200 and the consensus tree was obtained after summarizing the resulting topologies and discarding the “burn-in.” Trees from all 3 runs were combined into one file using LogCombiner while the consensus tree (Maximum Clade Credibility Tree) was built using TreeAnnotator (both applications distributed with BEAST).

### Index calculation and statistics

For each species, we obtained phylogenetic data alongside survivorship and behavioral data from the experiments. All analyses were performed using R v. 4.2.3 (R Core Team 2023).

To characterize the lifespans of workers within each species, we constructed a Kaplan–Meier plot, identified the maximum time for which all species had a non-zero survival probability, and calculated this probability beyond that time for each species (“survival” and “survminer” packages; Therneau 2020; Kassambara et al. 2021). This survivorship index was used in subsequent analysis.

To derive an informative summary variable capturing the behavior of each species during the tests, we conducted a principal component analysis (PCA) using the data from 4 measured behaviors: contact, digging around the victim, pulling the victim’s body parts, and biting the nylon thread. We generated 95 % confidence ellipses around centroids (means) from the PCA for the species. The PC1 means were then extracted as the helpfulness index and employed in subsequent analysis. We utilized the “ggcorrplot,” “ggbiplot,” “ggfortify,” and ‘factoextra’ packages to conduct the PCA (Vu 2011; Horikoshi and Tang 2016; Kassambara and Mundt 2020; Kassambara 2023).

To analyze the relationship between helpfulness and survivorship, taking into account the non-independence of data due to phylogenetic relatedness between the species, we used the phylogenetic generalized least squares model (PGLS) (Symonds and Blomberg 2014; Ravell and Harmon 2022). The model computed how the helpfulness index depended on the survivorship index and included the phylogenetic relationships in the variance-covariance matrix. *K* and λ were estimated to investigate the presence of phylogenetic signals in the 2 analyzed traits, the survivorship, and helpfulness indices. We utilized the “caper” package for PGLS (Orme et al. 2023).

## Results

**Fig. 1.**
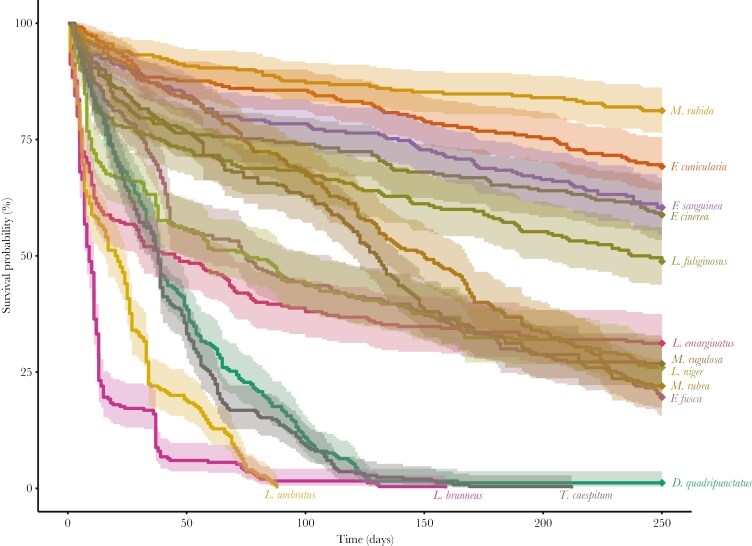
Kaplan–Meier survival plot for the species utilized in the study. Shadings indicate confidence intervals. Mortality was assessed for 5 colonies per species and 50 individuals from each colony over 250 d. The probability of worker survival beyond 80 d was used as the survivorship index and retained for further analyses.

The Kaplan–Meier plot visualizing mortality across species revealed that the maximum time for which all species had a non-zero survival probability was 80 d (Fig. 1). Thus, we calculated the survival probability of workers at 80 d in each species and retained it as the survivorship index (Supplementary Material 2).

**Fig. 2.**
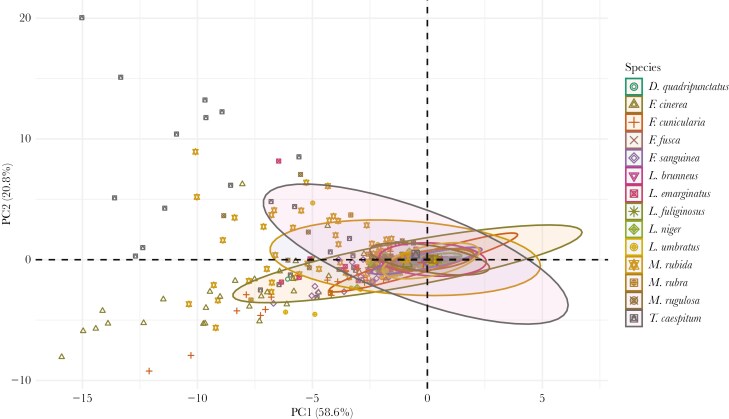
Principal component analysis score plot for the duration of contact with the victim, digging around it, pulling at its body parts, and biting the entrapping thread during behavioral rescue tests in each of the studied species. Shapes and colors differentiate species. Behavior was assessed for 5 colonies per species and 50 tests from each colony were performed. Ellipses indicate 95 % confidence intervals around means for the species and these means were used as the helpfulness index and retained for further analyses.

The first and second dimensions of the PCA for the measured behaviors accounted for nearly 80% of the variance. We extracted the helpfulness index for each species (Fig. 2). Behavior during the tests included contact, digging around the victim, pulling the victim’s body parts, and biting the nylon thread (see Supplementary Material 2). *M. rubida* demonstrated the highest mean contact rate (44 s). High standard deviation (SD) (57 s) suggested high variability within this species. Other species like *F. cinerea* and *T. caespitum* also showed notable contact, whereas species such as *L. fuliginosus* and *L. brunneus* had very low means (0.43 s and 2.2 s, respectively). *F. cinerea*, *M. rubida*, and *F. cunicularia* exhibited the highest digging activity (means of 4.4 s, 4.0 s, and 3.2 s, respectively). Several species (*D. quadripunctatus*, *L. brunneus*, *L. fuliginosus*) showed no digging behavior. *T. caespitum* and *F. cinerea* showed the highest mean pulling activity (3.8 s and 8.5 s, respectively), with relatively high SDs, indicating substantial intraspecies variability. Other species exhibited minimal or no pulling behavior. Thread biting was the least frequent behavior, with *T. caespitum* displaying the highest mean (3.7 s) and variability (SD = 15 s) (sporadic but intense thread biting). Most other species showed minimal or no thread-biting behavior, with the notable exception of *M. rubida*.

**Fig. 3.**
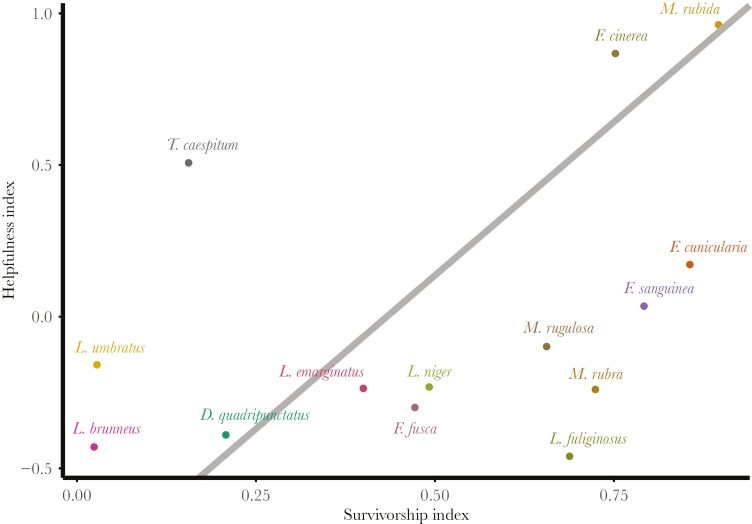
Helpfulness as a function of survival in the examined species. The helpfulness index is a summary variable derived from PCA analysis of four rescue behaviors (contact, digging, pulling, and biting) that indicates each species’ pro-social behavior during tests, while the survivorship index is calculated from a Kaplan–Meier plot that characterizes the lifespans of workers in each species, based on their survival probabilities over time. The gray solid line indicates the regression line estimated by the PGLS model.

The PGLS model demonstrated that the helpfulness index depended on the survivorship index (*F*_1,12_ = 10.50, *P* = 0.007, *R*^2^ = 0.467), with species characterized by higher worker life expectancies being more helpful toward nestmates (Fig. 3). There was no phylogenetic signal in either the survivorship index (*K* = 0.210, *P* = 0.098; λ = 0.502, *P* = 0.649) or the helpfulness index (*K* = 0.053, *P* = 0.791; λ < 0.001, *P* = 1).

## Discussion

Our results reinforce the idea that pro-social tendencies among Formicidae are associated with workers’ life expectancies. Specifically, longer-lived ant species are more helpful and more likely to take part in rescue actions toward nestmates than shorter-lived ant species. This result extends far beyond previous studies devoted to the effect of life expectancy on rescue behavior proneness (Miler 2016; Miler et al. 2017b; Frank et al. 2018; Beydizada et al. 2024). Miler (2016) demonstrated that *F. cinerea* ants with shortened life expectancy elicited lower levels of rescue behavior than healthy individuals. Another study showed that shortened life expectancy increased social withdrawal in this species (Miler et al. 2017b). Also, Frank et al. (2018) found that heavily injured *M. analis* ants (i.e. individuals with low life expectancy) did not receive help and were not treated after being placed in the nest in comparison to uninjured or less severely injured individuals. Heavily injured ants also showed social withdrawal, ignored their nestmates, and resisted being picked up and transported to the nest (Frank et al. 2018). Also, results on *Cataglyphis nodus* (Brullé, 1833) ants showed that leg injuries received more care than antennal injuries (Beydizada et al. 2024). When leg injuries became infected, care decreased and the injured ants were often expelled, highlighting a sophisticated social immunity strategy that differentiates between treatable and untreatable cases to protect the colony (Beydizada et al. 2024). Life expectancy is one of the most important factors driving the behavior of social insects, particularly ants, on a within-species scale (Woyciechowski and Kozłowski 1998; Tofilski 2002). As we show here, this is the case as well on a between-species scale, at least in terms of pro-social tendencies. Future research must investigate whether species with varying helpfulness, particularly rescue proneness, differ in fitness outcomes during ecologically relevant situations, such as nest collapse. This could provide novel, direct evidence for an ant colony as a higher-order unit of selection compared to the individual (Queller and Strassmann 1998; Helanterä et al. 2009).

In species of ants with relatively low worker life expectancy rescue actions were undetected or infrequent. In turn, species with relatively high worker life expectancy demonstrated increased proneness for helpfulness. Importantly, neither life expectancies nor helpfulness of workers showed phylogenetic constraints. A plausible explanation for this pattern is that the 2 traits coevolved independently many times, with a direct effect of one on the other (Carey 2001). Specifically, more pro-social behavior can lead to the care of other individuals within the colony and help in maintaining physical health and thus increase life expectancy. Additionally, this pattern might also reflect a high colony-level cost to saving members of the colony with relatively low life expectancies. In ant colonies, resources and energy are allocated to ensure the survival and high productivity of the entire colony (Hölldobler and Wilson 1990). Investing resources in interacting with and saving individuals that die soon anyway might be unproductive, especially if the energy spent on such risky and costly behavior could be directed toward tasks that contribute more significantly to the overall success of the colony, e.g. foraging (Bar et al. 2023). Indeed, Frank et al. (2017) found that *M. analis* ants rescued 9 to 15 workers per day and most of those saved (around 95%) participated in future colony activities, mitigating the need to replace them with new workers. Further exploration of the trade-offs and costs associated with rescue is essential to fully understand this phenomenon.

Our study offers an investigation into the life expectancy parameter under controlled conditions across species representing diverse phylogenetic affiliations. It shows significant differences in the life expectancy of foragers among various ant species. Kwon et al. (2022) found that the forager mortality rate due to interspecific competition in the field is higher in small-bodied, submissive, and subordinate species than in large-bodied, aggressive, and dominant species. An interesting question for future research is how environments of varying riskiness shape worker life expectancy among ants. Although such an investigation is beyond the scope of the current study, our results suggest that life expectancy might be interrelated to other factors, such as body size or dominance. Specifically, species that lived the longest were bigger and more dominant than those that lived for the shortest periods (Hölldobler and Wilson 1990; Czechowski et al. 2012). Additionally, this similarity between our results and those of Kwon et al. (2022) adds ecological relevance to our measurements of survivorship which, even without extrinsic causes of mortality, revealed similar mortality patterns as in the field (de Vries et al. 2023). It is worth noting that among the 14 species studied, several are dendrobionts or deep-soil dwellers, including *D. quadripunctatus*, *L. brunneus*, *L. fuliginosus*, and *L. umbratus* (Czechowski et al. 2012). Notably, 3 of these species, excluding *L. fuliginosus*, exhibited reduced life expectancy, likely due to substantial differences between laboratory conditions and their natural environments. Thus, in future research, we recommend incorporating substrates and key colony components, such as queens and brood, to better simulate natural conditions when assessing survival.

Rescue behavior has been observed in several distinct subfamilies, including Dolichoderinae, Formicinae, Myrmicinae, and Ponerinae (e.g. Czechowski et al. 2002; Hollis and Nowbahari 2013; Miler 2016; Frank et al. 2017; Miler et al. 2017a; Uy et al. 2019). Thus, it is clear that rescue occurs in distantly related ant species, which makes it plausible that this behavior evolved many times (Miler and Turza 2021). Indeed, we did not find a strong phylogenetic signal for this trait, indicating that it is phylogenetically labile and not constrained in its evolution. However, only 23 species have been studied for such behavior out of over 16 000 known species of ants so far (Bolton World Catalog 2024). It is worth pointing out that in our study, 11 out of 14 species were tested for the first time in the context of rescue behavior. Our data indicate that the rescue behavior of the tested species varies from pronounced to weak. Specifically, certain behaviors like contact and digging are more widespread across species, whereas pulling and thread biting are less common and more species-specific. The results are directly in line with previous findings suggesting that different species of ants represent different levels of rescue (Hollis and Nowbahari 2013; Miler et al. 2017a). Our results represent a substantial contribution to our understanding of pro-sociality within this group of insects. Future research should further develop this comprehensive approach. To date, most research questions regarding rescue behavior were addressed only in 2 species of ants, *Cataglyphis piliscapa* (Forel, 1901) (formerly *C*. *cursor* (Fonscolombe, 1846)) and *F. cinerea* (e.g. Nowbahari et al. 2009; Miler 2016; Duhoo et al. 2017; Turza et al. 2020). It is unclear how generalizable are the results obtained solely for these species. Already for these 2 species, we can find conflicting evidence in the available literature. For example, in the case of *C. piliscapa*, the most common type of rescue behavior is digging around the victim (Duhoo et al. 2017), while in *F. cinerea* ants it is pulling the victim (Turza and Miler 2023). The differences in the rescue actions of different ant species may suggest that these actions evolved in various contexts, depending on the selective pressure of the environment and ecology specific to a given species (Hollis and Nowbahari 2013). This underlines the necessity of investigating this phenomenon in species that have not been explored so far.

Some studies suggest that rescue behavior can depend on the ecology of the species in terms of nesting habitat (Hangartner 1969; Uy et al. 2019). For example, more frequent rescue behavior was observed in sand-dwelling ants (*C. floricola* Tinaut, 1993 and *C. piliscapa*) than in those living in more compact soils, like *Messor barbarus* (Linnaeus, 1767) and *M. marocanus* (Santschi, 1927) ants (Hollis and Nowbahari 2013). In our study, ant species characteristic for sandy and sunny habitats (i.e. *F. cinerea*, *M. rubida*, and *T. caespitum*) were indeed characterized by a high level of helpfulness. The high variability across species and behaviors that we detected suggests different ecological roles or interaction strategies. Expanding the number of studied species is crucial for definitive results in terms of the effect of species ecology (Miler and Turza 2021).

## Conclusions

In summary, deciphering how proximate and ultimate factors modulate pro-sociality is one of the central questions in animal behavior research (e.g. Cronin 2012; Marshall-Pescini et al. 2016; Chen et al. 2023; Nafcha et al. 2023). Here, using ants, we showed that their inter-species variation in pro-social tendencies, indicated by helpfulness, can be in part explained by life expectancy. Specifically, ant species characterized by higher worker life expectancies are more helpful toward nestmates. This pattern is similar to that observed in mammals. The effect of age on pro-social behavior has been demonstrated in wild primates (Lange et al. 2023). Also, more social species live longer, supporting the correlated evolution of social organization and longevity in the animal kingdom (Zhu et al. 2023; Salguero-Gómez 2024). We postulate that life expectancy should be considered more frequently as part of various behavioral patterns among group-living animals. The incorporation of this factor can provide a deeper understanding of the mechanisms by which all societies operate, from ants to primates.

## Supplementary Material

arae104_suppl_Supplementary_Materials_S1

arae104_suppl_Supplementary_Materials_S2

## Data Availability

Analyses reported in this article can be reproduced using the data provided by Turza et al. (2024).
