# Supplementary material for: Life expectancy in ants explains variation in helpfulness regardless of phylogenetic relatedness
Source: Behav Ecol. 2024 Dec 17;36(3):arae104. doi: 10.1093/beheco/arae104 (PMC11932148; doi:10.1093/beheco/arae104)
Supplement: arae104_suppl_Supplementary_Materials_S1 [file arae104_suppl_supplementary_materials_s1.zip › arae104_suppl_Supplementary_Materials_1/SM1.pdf]

Life expectancy in ants explains variation in helpfulness regardless of phylogenetic relatedness

Filip Turza[1,2], Daniel Stec[3], Diego Fontaneto[4,5], Krzysztof Miler[3]

[1] Doctoral School of Exact and Natural Sciences, Jagiellonian University, prof. S. Łojasiewicza 11, 30-348 Kraków, Poland

[2] Institute of Environmental Sciences, Faculty of Biology, Jagiellonian University, Gronostajowa 7, 30-387 Kraków, Poland

[3] Institute of Systematics and Evolution of Animals, Polish Academy of Sciences, Sławkowska 17, 31-016 Kraków, Poland

[4] Molecular Ecology Group (MEG), National Research Council of Italy, Water Research Institute (CNR-IRSA), Largo Tonolli 50, Verbania Pallanza, Italy

[5] National Biodiversity Future Center (NBFC), Piazza Marina 61, Palermo, Italy

Correspondence:

Filip Turza, Institute of Environmental Sciences, Faculty of Biology, Jagiellonian University, Gronostajowa 7, 30-387 Kraków, Poland. E-mail: filip.turza@uj.edu.pl

Krzysztof Miler, Institute of Systematics and Evolution of Animals, Polish Academy of Sciences, Sławkowska 17, 31-016 Kraków, Poland. E-mail: miler@isez.pan.krakow.pl

	Species	28S rRNA	COI
	Dolichoderus quadripunctatus	OR856564	OR856592
	Dolichoderus quadripunctatus	OR856565	OR856593
	Myrmica rubra	OR856586	OR856614
	Myrmica rubra	OR856587	OR856615
	Myrmica rugulosa	OR856588	OR856616
	Myrmica rugulosa	OR856589	OR856617
	Manica rubida	OR856584	OR856612
	Manica rubida	OR856585	OR856613
	Tetramorium caespitum	OR856590	OR856618
	Tetramorium caespitum	OR856591	OR856619
	Formica fusca	OR856570	OR856598
	Formica fusca	OR856571	OR856599
	Formica cinerea	OR856566	OR856594
	Formica cinerea	OR856567	OR856595
	Formica cunicularia	OR856568	OR856596
	Formica cunicularia	OR856569	OR856597
	Formica sanguinea	OR856572	OR856600
	Formica sanguinea	OR856573	OR856601
	Lasius niger	OR856580	OR856608
	Lasius niger	OR856581	OR856609
	Lasius emarginatus	OR856576	OR856604
	Lasius emarginatus	OR856577	OR856605
	Lasius brunneus	OR856574	OR856602
	Lasius brunneus	OR856575	OR856603
	Lasius fuliginosus	OR856578	OR856606
	Lasius fuliginosus	OR856579	OR856607
	Lasius umbratus	OR856582	OR856610
	Lasius umbratus	OR856583	OR856611
OUTGROUP	*Paraponera clavata*	DQ353641	DQ353276

Supplementary Table 1. Sequence accession numbers submitted to GenBank.
