# Supplementary material for: Life expectancy in ants explains variation in helpfulness regardless of phylogenetic relatedness
Source: Behav Ecol. 2024 Dec 17;36(3):arae104. doi: 10.1093/beheco/arae104 (PMC11932148; doi:10.1093/beheco/arae104)
Supplement: arae104_suppl_Supplementary_Materials_S1 [file arae104_suppl_supplementary_materials_s1.zip › arae104_suppl_Supplementary_Materials_1/supplementary material1.docx]

**Supplementary Materials**

Supplementary Material 1. Sequence accession numbers submitted to GenBank.
