# Supplementary material for: Life expectancy in ants explains variation in helpfulness regardless of phylogenetic relatedness
Source: Behav Ecol. 2024 Dec 17;36(3):arae104. doi: 10.1093/beheco/arae104 (PMC11932148; doi:10.1093/beheco/arae104)
Supplement: arae104_suppl_Supplementary_Materials_S2 [file arae104_suppl_supplementary_materials_s2.zip › arae104_suppl_Supplementary_Materials_2/SM2.pdf]

Life expectancy in ants explains variation in helpfulness regardless of phylogenetic relatedness

Filip Turza[1,2], Daniel Stec[3], Diego Fontaneto[4,5], Krzysztof Miler[3]

[1] Doctoral School of Exact and Natural Sciences, Jagiellonian University, prof. S. Łojasiewicza 11, 30-348 Kraków, Poland

[2] Institute of Environmental Sciences, Faculty of Biology, Jagiellonian University, Gronostajowa 7, 30-387 Kraków, Poland

[3] Institute of Systematics and Evolution of Animals, Polish Academy of Sciences, Sławkowska 17, 31-016 Kraków, Poland

[4] Molecular Ecology Group (MEG), National Research Council of Italy, Water Research Institute (CNR-IRSA), Largo Tonolli 50, Verbania Pallanza, Italy

[5] National Biodiversity Future Center (NBFC), Piazza Marina 61, Palermo, Italy

Correspondence:

Filip Turza, Institute of Environmental Sciences, Faculty of Biology, Jagiellonian University, Gronostajowa 7, 30-387 Kraków, Poland. E-mail: filip.turza@uj.edu.pl

Krzysztof Miler, Institute of Systematics and Evolution of Animals, Polish Academy of Sciences, Sławkowska 17, 31-016 Kraków, Poland. E-mail: miler@isez.pan.krakow.pl

Supplementary Table 1. Full results of the survival probability of workers at 80 days in each species.

Species	Survival probability	Std. Err.	Lower 95% CI	Upper 95% CI
Manica rubida	0.896	0.0193	0.859	0.935
Formica cunicularia	0.856	0.0222	0.814	0.901
Formica sanguinea	0.792	0.0257	0.743	0.844
Formica cinerea	0.752	0.0273	0.7	0.807
Myrmica rubra	0.724	0.0283	0.671	0.782
Lasius fuliginosus	0.688	0.0293	0.633	0.748
Myrmica rugulosa	0.656	0.03	0.6	0.718
Lasius niger	0.492	0.0316	0.434	0.558
Formica fusca	0.472	0.0316	0.414	0.538
Lasius emarginatus	0.4	0.031	0.344	0.466
Dolichoderus quadripunctatus	0.208	0.0257	0.163	0.265
Tetramorium caespitum	0.156	0.0229	0.117	0.208
Lasius umbratus	0.028	0.0104	0.0135	0.0581
Lasius brunneus	0.024	0.00968	0.0109	0.0529

Supplementary Table 2. Descriptive statistics of particular rescue behaviors from 250 tests per species.

Species	Contact Mean	Contact Std.Dev.	Digging Mean	Digging Std.Dev.	Pulling Mean	Pulling Std.Dev.	Thread biting Mean	Thread biting Std.Dev.
Manica rubida	44	57	4	12	3.4	9.3	2.8	7.9
Formica cunicularia	18	31	3.2	14	2.2	10	0.024	0.38
Formica sanguinea	22	35	1.5	6.8	0.32	2.6	0	0
Formica cinerea	32	56	4.4	14	8.5	27	0.54	3.4
Myrmica rubra	9.1	20	0.54	3.7	0.4	4	0.14	1.6
Lasius fuliginosus	0.43	2.9	0.012	0.19	0	0	0	0
Myrmica rugulosa	14	30	0.27	1.3	1.3	6.6	0.52	3.6
Lasius niger	10	19	0.63	3.1	0.19	0.82	0.068	0.53
Formica fusca	8.6	20	0.13	1.2	0.15	1	0.016	0.25
Lasius emarginatus	7.3	24	0.28	1.9	1.3	7.1	0.26	3.3
Dolichoderus quadripunctatus	3.4	11	0	0	0.38	5.3	0	0
Tetramorium caespitum	23	47	1.6	5.8	3.8	17	3.7	15
Lasius umbratus	8.6	18	2	8	0.16	0.83	0.2	2
Lasius brunneus	2.2	5.8	0.004	0.063	0.004	0.063	0.004	0.063
