# Supplementary material for: Life expectancy in ants explains variation in helpfulness regardless of phylogenetic relatedness
Source: Behav Ecol. 2024 Dec 17;36(3):arae104. doi: 10.1093/beheco/arae104 (PMC11932148; doi:10.1093/beheco/arae104)
Supplement: arae104_suppl_Supplementary_Materials_S2 [file arae104_suppl_supplementary_materials_s2.zip › arae104_suppl_Supplementary_Materials_2/supplementary material2.docx]

**Supplementary Materials**

Supplementary Material 2. Survival probability in each species and descriptive statistics for the duration of contact with the victim, digging around it, pulling at its body parts, and biting the entrapping thread during behavioral rescue tests in each of the studied species.
